# Enigmatic Rapidly Enlarging Nasal Mass That Is Not Cancer

**DOI:** 10.1155/2020/8855572

**Published:** 2020-08-26

**Authors:** Trilok Shrivastava, Lin Li, Sonia Tanwar, Rabab Nasim, Vincent Gullas, Jay Pescatore, Michael Nelson

**Affiliations:** ^1^Department of Internal Medicine, John H Stroger Hospital of Cook County, Chicago, IL, USA; ^2^Emergency Department, John H Stroger Hospital of Cook County, Chicago, IL, USA

## Abstract

Cutaneous blastomycosis is not rare, but progressively enlarging nasal mass as the only presentation with nondiagnostic biopsy results, presence of pulmonary fibrosis, nodules and lymphadenopathy, and urinary sediments, as well as ANA and p-ANCA positivity, can make things more cryptic than expected.

## 1. Introduction

A variety of conditions can lead to deforming skin lesions involving the nose which include granulomatous diseases, vasculitides, fungal infections, atypical bacteria, and neoplasms, but only a few can be rapidly progressing and have systemic involvement. In this report, we present an elderly Hispanic lady who lives in Chicago, Illinois, with no recent travel history presenting with a distorting nasal mass that had been enlarging over a duration of 4 months and accelerated over a period of 2 weeks. She was also found to have left submandibular lymphadenopathy, bilateral pulmonary nodules, hilar and mediastinal lymphadenopathy, and fibrotic pulmonary opacities. The course of investigation was some-what twisted with ANA and p-ANCA positivity, and initial punch biopsy of primary nasal mass was unrevealing but subsequent biopsy being able to demonstrate findings consistent with Blastomyces, reinforced by positive urine Blastomyces antigen.

Blastomycosis is a systemic granulomatous disease caused in majority by *Blastomyces dermatitidis* and *Blastomyces gilchristii* which are dimorphic fungi endemic to North American regions close to Mississippi and Ohio River basins, the Great Lakes, and St. Lawrence River. Transmission is through inhalation of aerosolized conidia leading to a wide range of system involvement [1, 2]. While a majority of cases present with respiratory problems, there have been quite a few presentations with primary extrapulmonary manifestations causing diagnostic dilemma [3–6].

## 2. Case History and Examination

**Figure 1:**
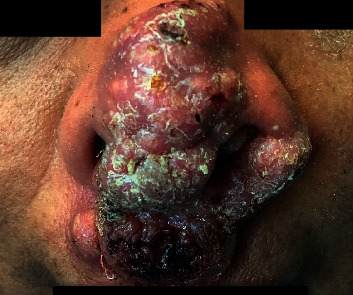
Nasal mass during admission showing involvement of left ala, nasal bridge, columella, philtrum and left nasal cavity with irregular scaly micronodular lesion with pustules, and areas of hemorrhage.

**Figure 2:**
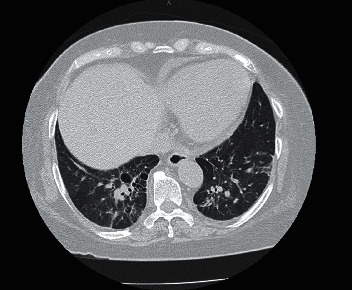
CT chest showing fibrotic parenchyma of both lungs more on the right side with nodules.

A 69-year-old lady with a past medical history of diabetes, hypertension, stage-4 chronic kidney disease, and systolic heart failure presented with a rapidly growing nasal mass over a period of 4 months that started out as a pimple which ruptured and started to grow from the nasal bridge up to the tip of nose along columella interiorly into her left nasal cavity. She denied pain, fever, cough, dyspnea, gastrointestinal symptoms, chest pain, visual abnormalities, hematuria, or recent travel. During her admission 3 months back for an episode of decompensated heart failure, the mass was noted to be small, so she was referred to an ENT clinic where a superficial biopsy was done. The histopathology result was nondiagnostic showing squamous mucosa with pseudoepitheliomatous hyperplasia along with acute and chronic inflammatory changes. After this biopsy, the mass enlarged rapidly causing nasal congestion and runny nose, and the patient was admitted to the hospital for further evaluation of the mass. Patient was afebrile throughout admission, and her hemodynamics were stable. Examination showed a large exophytic left nasal mass involving the nasal bridge, columella, ala, philtrum with crusting, areas of hemorrhage, and a few pustules. Left nostril was almost completely occluded during the end of her hospital admission. There was also a 2 cm mobile mildly tender left submandibular lymphadenopathy. Lung exam was unremarkable (Figures 1 and 2).

### 2.1. Differential Diagnosis, Investigations, and Treatment

CT neck confirmed the examination findings and also demonstrated inferior nasal turbinate connection of the mass, while CT of the chest showed bilateral pulmonary nodules, bilateral fibrotic opacities, pleural thickening of bilateral pleura, fibrocystic changes of the right lower lobe of the lung, and posterior mediastinal and probable hilar lymphadenopathy. A repeat biopsy of the nasal lesion was again inconclusive demonstrating nonspecific chronic inflammation, fibrosis, and suppurative necrosis. Special stains were negative for fungal infections and acid-fast bacilli. During this time, the differential diagnoses were granulomatous and inflammatory diseases such as sarcoidosis, small vessel vasculitis viz. granulomatosis with polyangiitis, tuberculosis, atypical fungal infections such as rhinosporidiosis, histoplasmosis, blastomycosis, and malignancy; therefore, multidisciplinary discussion was done involving pulmonology, rheumatology, ENT, infectious disease, nephrology, dermatology, and cardiology. On laboratory evaluation, she was noted to have chronic stable anemia with normal white counts, and liver profile was normal except for mild hypoalbuminemia. Her autoimmune panel came back with positive antinuclear antibody (ANA) (<1 : 160) and myeloperoxidase antineutrophil cytoplasmic antibody (MPO-ANCA/p-ANCA) with a titer of 7.6 (ref.: <1). Anti-dsDNA, anti-Smith-Ab, anti-RNP, anti-SSA, and anti-SSB were negative, and C3 and C4 were within normal limits. ESR and CRP were elevated at 103 mm/hr and 8.6 mg/dl, respectively. Her previous urinalysis had few RBC casts, but recent urine was bland; therefore, renal biopsy was deferred. Three sputa smears for acid-fast bacilli, HIV and cryptococcal antigen, and hepatitis A, B, and C serologies were negative. Urine histoplasma and Blastomyces antigen were sent, but results are pending. The left nostril was packed with Merocel impregnated in dexamethasone, and the patient was covered with intravenous ampicillin-sulbactam for possible bacterial superinfection. Bronchoscopy with a biopsy of accessible hilar lymph node was considered, but patient refused the procedure; so, it was withheld. Therefore, a repeat biopsy of the nasal mass and submandibular lymph node was done, and the specimen was sent for special fungal stains, AFB cultures, and histopathology.

The patient was discharged with symptomatic care after the repeat punch biopsy of the nose and cervical lymph node with pending results. Urine antigen came back positive for both Histoplasma and Blastomyces antigens, and the third biopsy demonstrated noncaseating granulomatous inflammation with fungal elements that, on staining with periodic acid-Schiff (PAS) and Grocott-Gomori methenamine silver nitrate (GMS) stains, it showed large round to oval yeasts with double contour and single broad-based buds consistent with blastomycosis. Putting together her clinical picture and laboratory findings, she was diagnosed to have disseminated blastomycosis.

### 2.2. Outcome and Follow-Up

The patient is being readmitted for systemic antifungal therapy.

## 3. Discussion

Blastomycosis can present with a wide range of symptoms, and cutaneous manifestations are common representing 40–80% of the cases subdivided into verrucous and ulcerative forms; cases presenting with facial verrucous plaques have been reported [2, 3, 7–9].

What made this case difficult to diagnose was the paucity as well as diversity of pathognomic dermatological findings to any disease; initially, negative biopsy result and positive ANA & MPO-ANCA diverted it towards a vasculitic process, reinforced by presence of RBC casts in one of her previous urinalyses. While granulomatosis with polyangiitis (GPA) is typically associated with c-ANCA/PR3-ANCA, nearly 20% have MPO-ANCA, although the nasal dermatological findings are usually limited to nasal crusting, saddle nose deformity, septal perforation, and sanguineous nasal discharge in these patients and not as extensive as those seen in our patient [10, 11].

Sarcoidosis was another consideration in this patient given her pulmonary nodules, hilar and mediastinal lymphadenopathy, and fibrocystic changes of pulmonary parenchyma. Had the patient allowed bronchoscopy with bronchoalveolar lavage and transbronchial lymph node biopsy, we might have excluded sarcoidosis if the CD4/CD8 ratio was <1 : 1, but if we only found noncaseating granuloma with the ratio in between 1 : 1–4 : 1, it would still be indeterminate, but fungal stains might have still been of essence in that case [12, 13].

Up to 30% of facial basal cell carcinomas (BCC) are found in the nose, and they can invade and obliterate the neighboring anatomy [14]. Squamous cell carcinoma (SCC) is much less common to involve the nose, although possible, and among them, most neoplasms are keratinizing squamous cell carcinomas that usually originate from the sinonasal tract [15]. There has also been a report of lung cancer metastasizing to the nose with “clown nose” as the presenting symptom [16]. However, three sets of biopsies were done in this patient which ruled out any of these malignancies.

Interestingly, the initial skin biopsy results in our patient were similar to the findings in few other reported cases of cutaneous blastomycosis which also displayed pseudoepitheliomatous hyperplasia, an entity that itself represents inflammatory, infectious, and neoplastic conditions [9, 17, 18]. The American Society of Microbiology recommends searching for yeast forms in the biopsy specimens with GMS staining to diagnose blastomycosis, which in fact helped to discover the broad-based buds of Blastomyces in our patient [2]. Her urine antigen positivity for Histoplasma most likely accounts for the cross-reactivity among these two pathogens, which occurs in about 70% of cases [19].

The Infectious Disease Society of America (IDSA) recommends to treat patients with moderate-severe disseminated extrapulmonary blastomycosis as our patient with lipophilic amphotericin B for 1-2 weeks or until clinical improvement that must be followed by oral itraconazole at a tapering-maintenance dose for a total duration of 12 months [20].
